# Modulation of the Tumor Microenvironment by Ellagic Acid in Rat Model for Hepatocellular Carcinoma: A Potential Target against Hepatic Cancer Stem Cells

**DOI:** 10.3390/cancers15194891

**Published:** 2023-10-09

**Authors:** Wafaa S. Ramadan, Saleh Alkarim, Mohammed Moulay, Ghadeer Alrefeai, Fatma Alkudsy, Khalid Rehman Hakeem, Ashwaq Iskander

**Affiliations:** 1 Department of Clinical Anatomy, Faculty of Medicine, King Abdulaziz University, Jeddah 22384, Saudi Arabia; wramadhan@kau.edu.sa; 2 Embryonic Stem Cell Research Unit, King Fahd Medical Research Center, King Abdulaziz University, Jeddah 21589, Saudi Arabia; 3 Embryonic and Cancer Stem Cell Research Group, King Fahd Medical Research Center, King Abdulaziz University, Jeddah 21589, Saudi Arabia; 4 Department of Biological Science, Faculty of Sciences, King Abdulaziz University, Jeddah 21589, Saudi Arabia; 5 King Fahd Medical Research Center, King Abdulaziz University, Jeddah 21589, Saudi Arabia; 6 Department of Biology, Abdelhamid ibn Badis University, Mostaganem 27000, Algeria; 7 Department of Biology, Faculty of Science, University of Jeddah, Jeddah 23218, Saudi Arabia; 8 Princess Dr. Najla Bint Saud Al-Saud Center for Excellence Research in Biotechnology, King Abdulaziz University, Jeddah 21589, Saudi Arabia; 9 Department of Public Health, Daffodil International University, Dhaka 1341, Bangladesh

**Keywords:** CSCs, HCC rat model, TGF alpha, TGF beta, VEGF, apoptosis, p53

## Abstract

**Simple Summary:**

The challenges for chemotherapeutic treatment in hepatocellular carcinoma (HCC) are high due to drug resistance or relapse. Such durability was attributed to the presence of hepatic cancer stem cells (HCSCs). In this study, we evaluate the therapeutic effect of ellagic acid (EA), a phytochemical, against HCC in a rat model induced by CCL4 and further investigate the reaction of the HCC microenvironment and HCSCs in response to systematic EA therapy.

**Abstract:**

The resistance to therapy and relapse in hepatocellular carcinoma (HCC) is highly attributed to hepatic cancer stem cells (HCSCs). HCSCs are under microenvironment control. This work aimed to assess the systemic effect of ellagic acid (EA) on the HCC microenvironment to decline HCSCs. Fifty Wistar rats were divided into six groups: negative control (CON), groups 2 and 3 for solvents (DMSO), and (OVO). Group 4 was administered EA only. The (HCC-M) group, utilized as an HCC model, administered CCL4 (0.5 mL/kg in OVO) 1:1 *v*/*v*, i.p) for 16 weeks. HCC-M rats were treated orally with EA (EA + HCC) 50 mg/kg bw for five weeks. Biochemical, morphological, histopathological, and immunohistochemical studies, and gene analysis using qRT-PCR were applied. Results revealed elevated liver injury biomarkers ALT, AST, ALP, and tumor biomarkers AFP and GGT, and marked nodularity of livers of HCC-M. EA effectively reduced the biomarkers and restored the altered structure of the livers. At the mRNA level, EA downregulated the expression of TGF-α, TGF-β, and VEGF, and restored p53 expression. This induced an increase in apoptotic cells immunostained with caspase3 and decreased the CD44 immunostained HCSCs. EA could modulate the tumor microenvironment in the HCC rat model and ultimately target the HCSCs.

## 1. Introduction

Only a few carcinomas were reported to attain long-term survival and respond to therapeutic measures without resistance or relapse after initial treatment [1]. In hepatocellular carcinoma (HCC), the challenges for the treatment are high; only 5% to 15% of early-stage patients can be subjected to surgical hepatectomy, intermediate stages can undergo transarterial chemoembolization with the hope of a 2-year survival rate [2]. Such durability was attained in the presence of cancer cells that resist therapy and death by specific microenvironment signals [3]. 

Cancer stem cells (CSCs) were proposed to self-renew, survive, and migrate to distinct body sites [4] and thus be responsible for resistance and relapse [5]. CSCs were proven to interact with the tumor microenvironment to maintain stemness. The hypoxic microenvironment, acidity, and persistent inflammation induced CSC activation and aggressiveness [6,7]. 

Inflammation was linked to an increased probability of malignant tumors, carcinogenesis, metastasis, angiogenesis, anti-apoptosis, epigenetic modifications, and boosting proliferation [8,9,10]. The development of HCC was proved to be preceded by inflammation as more than 90% of HCC cases were on top of chronic inflammatory liver injury [11]. Modulating the inflammatory microenvironment of HCC could be a therapeutic target. 

Tumor suppressor genes as P53 control CSC stemness [12]. Similarly, proinflammatory cytokines and growth factors are essential in maintaining inflammation and promoting tumor formation [7]. Therefore, targeting CSCs can break the vicious circle of resistance to therapy and tumor relapse, hoping for long-term survival.

Phytomedicine gained attention for treating several diseases known for their resistance to therapy [13]. Ellagic acid (EA) (4,4′,5,5′,6,6′-Hexahydroxydiphenic acid 2,6,2′,6′-dilactone), a natural polyphenolic compound, is found in some fruits like berries and pomegranate. EA proved to be an antioxidant, antifibrotic, and anticarcinogenic agent [14]. EA was proven to mitigate the angiogenesis process, characteristic of carcinoma formation, involving endothelial cell proliferation and migration [15]. Other studies confirmed that EA can augment cell proliferation and enhance apoptosis in hormonal-independent prostate cancer cell lines [16] and in HeLa cells [17].

These mechanisms were also evident in vivo where EA induced apoptosis and halted the cell cycle in the G1 phase in the colon carcinoma rat model [18].

Also, the anti-inflammatory effect of EA was demonstrated in a previous study implemented in lung cancer induced in a rat model. EA decreased the expression of NF-κB and pro-inflammatory cytokine TNF-α [19].

EA has shown an immune-regulating capacity as it effectively enhances the release of innate immune mediators [20]. The tumor immune microenvironment has proved to play a pivotal role in advanced tumor therapy [21].

The present work was organized to explore the chemotherapeutic effect of systemic EA against HCC in a rat model induced by CCL4 and further explore the reaction of the HCC microenvironment and HCSCs in response to systematic therapy.

## 2. Materials and Methods

### 2.1. Animals and Experimental Design

Fifty adult male Wistar rats with an average weight of 180–200 g were placed in plastic cages (40.5 × 25 × 15 cm) and kept at 22 ± 1 °C and 60% humidity. Animal care was given as per the institutional guidelines for the care and use of laboratory animals by following the “Principles of Laboratory Animal Care”, and the declaration of Helsinki as amended in 1996. Approval from the ethical committee for animal research at the Faculty of Pharmacy at King Abdulaziz University REF. “PH-1443-45”. Intraperitoneal administration of sodium pentobarbital (50 mg/kg) was used to anesthetize the animals [22] for blood collection from the retro-orbital vein and dissection of the animals at the end of the experiment.

Animals were divided randomly into six groups as follows.

Group 1 (CON): Five rats were kept untreated to act as a negative control.
Group 2 (DMSO): Five rats were given the DMSO same quantity in which the EA was dissolved.
Group 3 (OVO): Five rats were administered olive oil, the same amount in which the CCL4 was dissolved.
Group 4 (EA): Five rats were administered EA at a dose of 50 mg/kg b.wt dissolved in DMSO following manufacturer laboratory procedures.
Group 5 (HCC-M): Thirty rats were used for induction of HCC by a single dose of CCl4 (0.5 mL/kg *i.p* in olive oil) twice weekly for eight weeks. The duration was modified from that applied by [23] based on what was declared that the administration of a different amount (up to 0.5 mL/kg) might affect tumor numbers [24].
Group 6 (EA + HCC): Fifteen rats confirmed with HCC from group HCC-M were administered EA orally at 50 mg/kg bw [18] daily for five weeks.

Animals of all groups were sacrificed after 13 weeks from the start of the experiment.

### 2.2. Materials

#### 2.2.1. Chemicals

Ellagic acid was purchased from Cayman Chemical (Ann Arbor, MI, USA).

Carbon tetrachloride (CCL4), Dimethyl sulfoxide (DMSO) 99.9% pure solvent from Compass Laboratory (Memphis, TN, USA).

#### 2.2.2. Biochemical Analysis

Collected blood samples were left to coagulate. Sera was separated by centrifugation at 5000× *g* for 15 min and immediately stored at −80 °C. Liver enzymes, aspartate aminotransferase (AST), alanine aminotransferase (ALT), and alkaline phosphatase (ALP) were analyzed to assess liver functions. Alpha-fetoprotein (AFP) and gamma-glutamyl transferase (GGT) were evaluated as indicators of HCC development and considered tumor biomarkers.

#### 2.2.3. Morphology

By the end of the 13th week, animals were weighed and anesthetized, abdomens were dissected, and livers extracted, weighed, examined grossly, and photographed using a digital camera. Sections of the livers were fixed in 10% neutral-buffered formalin for further histological and immunohistochemical studies. The remaining liver tissues were stored at −80 °C for molecular analyses.

#### 2.2.4. Liver Index %

Liver index was calculated according to the formula [25];

liver weight/body weight × 100.

#### 2.2.5. Histopathology

Livers fixed in neutral buffered formalin were dehydrated, cleared, and further processed into paraffin blocks. The 4 µm cut sections were stained with hematoxylin and eosin (H&E) [26].

#### 2.2.6. Immunohistochemical Study

Cut sections of previously prepared paraffin blocks were deparaffinized for immunohistochemical staining with monoclonal mouse antibody for Caspase 3 (Beijing, China) and CD44 (Anti-CD44 antibody [OX49] (ab238464) Boston, MA, USA following the manufacturer protocol. Sections were photographed using OLYMPUS (DP73) (BX53, Tokyo, Japan). 

##### Image Analysis

Image-Pro Plus software (Media Cybernetics, Rockville, MD, USA) version 7 [27] was used to analyze images (magnification ×200) and measure the mean area % of areas positively immunostained with caspase three and CD44. Two independent observers examined five non-overlapping visual fields in five sections for each rat in a blinded fashion.

#### 2.2.7. Quantitative Real-Time PCR (qRT-PCR)

An RNA extraction kit was used to extract total RNA from the isolated rat livers (Bioer Technology, Hangzhou, China). The quantity and RNA purity was assessed using the NanodropTM spectrophotometer at the A260/A280 wavelength. The Revert Aid QuantiTect Reverse Transcription Kit (QIAGEN, Hong Kong, China) was utilized for cDNA synthesis. Following the manufacturer’s instructions, (qRT-PCR) was used to assess the expression of multiple genes, p53, TGF alpha and beta, and VEGF. The primers utilized in this study are displayed in Table 1. Glyceraldehyde-3-phosphate dehydrogenase (GAPDH) served as a model housekeeping gene. That was conducted in triplicate for each sample. Livak (2-CT) technique was used to calculate the gene expression in this experiment following the formula; Target amount = 2^−ΔΔCt^ where ΔΔCt = [Ct (target gene) Ct − (GAPDH)] − [Ct (control) − Ct (GAPDH control)]. The fold difference for gene expression was calculated as 2^−ΔΔCT^ using the endogenous control genes (liver). The identity and purity of the amplified product were evaluated by melting curve analysis.

##### Statistical Study

Data collected were analyzed using SPSS software (IBM-USA) version 20. Means were compared using ANOVA test and when equal variance could be assumed, LSD test was applied. Data were revealed as mean ± standard deviation.

## 3. Results 

### 3.1. Biochemical Analysis

**Figure 1:**
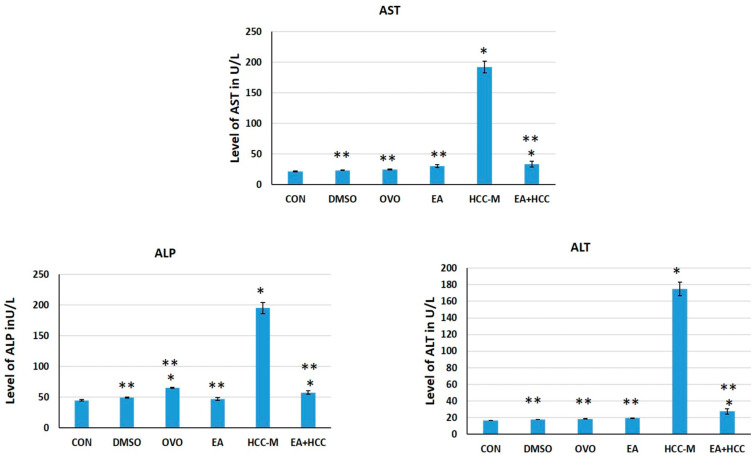
Bar graph showing levels of liver injury biomarkers, alanine aminotransferase (ALT) aspartate transaminase (AST), and alkaline phosphatase (ALP) in sera of all groups. Control (CON), (DMSO), olive oil (OVO), ellagic acid (EA), hepatocellular carcinoma model (HCC-M), and the HCC model treated with EA (EA + HCC). Data are presented as mean + SD. The mean difference was significant at level = 0.05; (**) significantly different from the HCC-M group at *p* ≤ 0.05; (*) significantly different from the CON group, *p* ≤ 0.05.

The enzymes concerning liver function, ALT, AST, and ALP were analyzed in the sera of animals of all groups. The enzymes were significantly increased in animals of group HCC-M compared to those in other groups, indicating the marked injurious effect of CCL4 on the liver (*p* ≤ 0.05) (Table 1). The introduction of EA as a therapeutic measure effectively decreased ALT, AST, and ALP levels, reflecting amelioration of the pathological status of the liver (Table 2) (Figure 1). 

**Figure 2:**
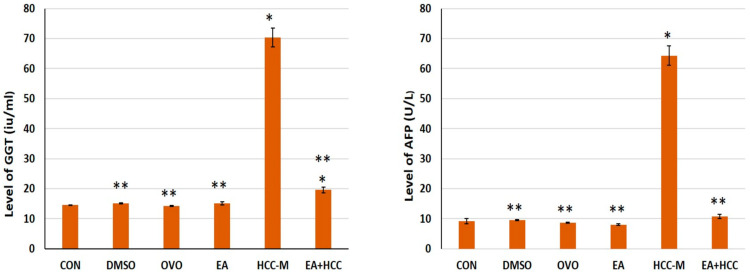
Bar graph showing levels of tumor markers, gamma-glutamyl transferase (GGT), and alpha fetoprotein (AFP) in sera of all groups. Control (CON), (DMSO), olive oil (OVO), ellagic acid (EA), hepatocellular carcinoma model (HCC-M), and the HCC model treated with EA (EA + HCC). Data are presented as mean + SD. The mean difference was significant at level = 0.05: (**) significantly different from the HCC-M group at *p* ≤ 0.05; (*) significantly different from the CON group, *p* ≤ 0.05.

Tracing the effect of CCL4, tumor markers AFP and GGT were analyzed in sera of animals. AFP was increased by 85.69% and GGT by 79.37% in the sera of animals of HCC-M compared to the CON group. This was highly indicative of HCC development. EA administration decreased levels of AFP by 83.26% and GGT by 72.17%, which implies the therapeutic effect of EA in cases of HCC (Figure 2).

#### 3.1.1. Morphological Study

**Figure 3:**
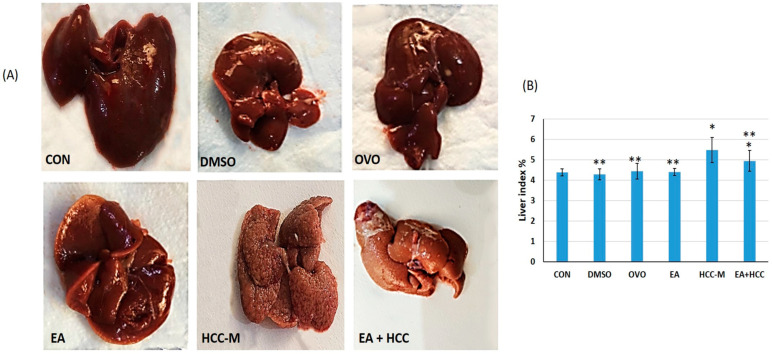
(**A**) Photomacrographs of livers from all groups. Control (CON), (DMSO), olive oil (OVO), ellagic acid (EA), hepatocellular carcinoma model (HCC-M), and the HCC model treated with EA (EA + HCC). A marked nodular surface of the HCC-M liver is evident which declined in the liver of group EA + HCC. (**B**) Bar graph showing the liver index % in all groups. Data are presented as mean + SD. The mean difference was significant at level = 0.05; (**) significantly different from the HCC-M group at *p* ≤ 0.05; (*) significantly different from the CON group, *p* ≤ 0.05.

A gross examination of the dissected livers of all groups revealed marked nodular surfaces of the livers of the HCC-M group. The nodularity was significantly reduced on treatment with EA in the livers of group EA + HCC (Figure 3A).

All groups’ weights of the livers were documented at the end of the experiment before the animals’ sacrifice. The liver index % was calculated and statistically analyzed. There was a significant increase in the liver index % of group HCC-M compared to all other groups (*p* ≤ 0.05). Such an increase responded to the therapeutic effect of EA, resulting in its reduction (Figure 3B).

#### 3.1.2. Histopathology

**Figure 4:**
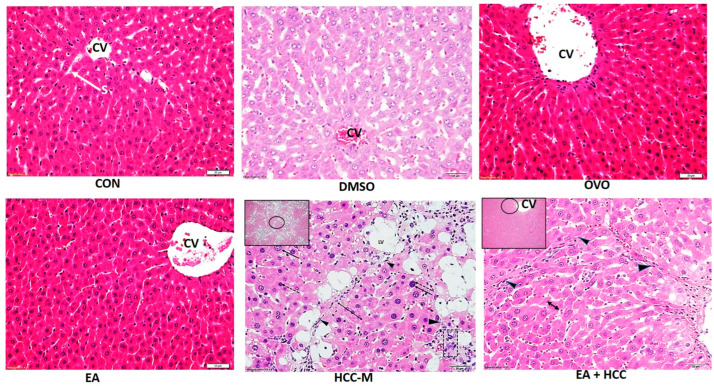
Light microscopic photomicrographs of sections of livers of all groups. Normal hepatic histological structure revealed in sections of groups, CON, DMSO, OVO, and EA administration of CCL4 in the group (HCC-M) revealed distorted structures with eminent nodular formation (arrowheads), hepatocytes ballooned with lipid vacuoles (LV), and arranged in plates of 2 cells (double arrow), nuclear hyperchromasia (dashed arrows). Increased nuclear-cytoplasmic ratio (arrows). Clusters of oval cells (square). In sections of livers from EA + HCC group, general hepatic architecture regained with hepatocyte plates arranged in one cell plate (double arrow) and the nodular pattern was still evident (arrowheads). CV = central vein. (H&E ×400). In cents (H&E ×100) scale bar = 20 µm.

The microscopic examination of liver sections stained with H&E revealed a typical structural pattern—hepatocytes with vesicular nuclei radiating in rows from the central vein separated by sinusoids. The structure of livers from rats of CON, DMSO, OVO, and EA groups was indistinguishable except for slight central vein (CV) congestion in the DMSO group. Livers from rats of group HCC-M revealed distorted structures with eminent nodular division. Hepatocytes ballooned with lipid vacuoles of different sizes and arranged in plates of more than two cells. Nuclei were prominent, hyperchromatosed with an apparent increased nuclear-cytoplasmic ratio. Clusters of oval cells were also evident. In sections of livers from the EA + HCC group, general hepatic architecture was regained with hepatocyte plates arranged in one cell plate, and the prominent fatty change was almost abolished. However, the nodular pattern was still evident (Figure 4).

#### 3.1.3. Immunohistochemical Study

**Figure 5:**
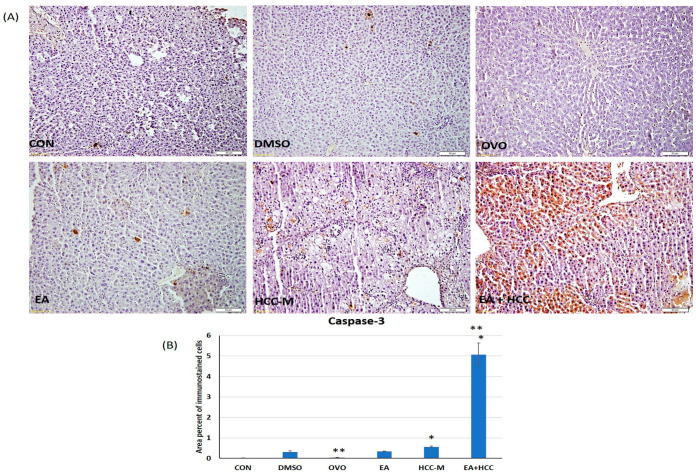
Photomicrograph of sections of livers immunostained with caspase 3 in different groups (**A**); control (CON), (DMSO), olive oil (OVO), ellagic acid (EA), hepatocellular carcinoma model (HCC-M), and the HCC model treated with EA (EA + HCC). Scale bar = 50 µm (**B**) bar graph representing the area percent of immunostained cells in all groups. Data are presented as mean + SD. The mean difference was significant at level = 0.05; (**) significantly different from the HCC-M group at *p* ≤ 0.05; (*) significantly different from the CON group, *p* ≤ 0.05.

In this study, cells immunostained with caspase-3 were used to identify the final execution of apoptosis. There was a remarkable increase in the area percent of immunostained cells in sections of livers of group EA + HCC (5.07 ± 0.55) compared to the untreated group HCC-M (0.55 ± 0.042). Other groups (DMSO, OLO, and EA) did not show any significant difference from the CON (*p* ≥ 0.05) (Figure 5A,B).

**Figure 6:**
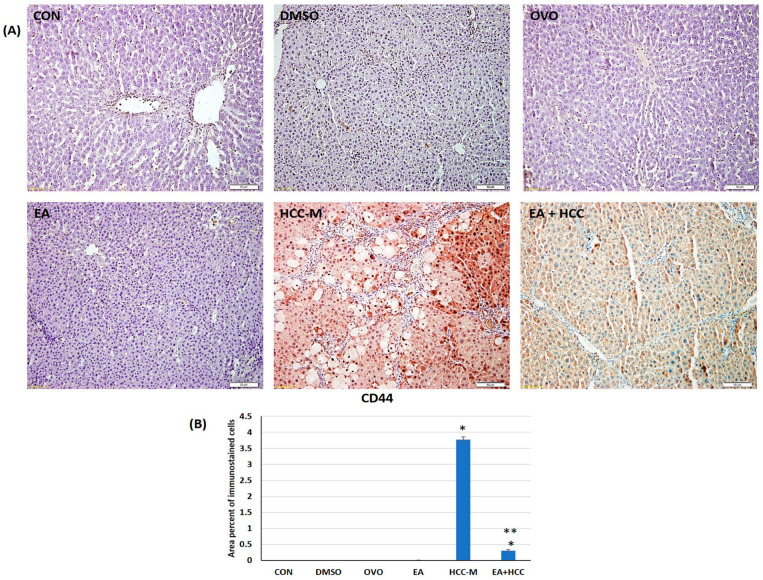
Photomicrograph of sections of livers immunostained with CD44 marking hepatic cancer stem cells (HCSCs) in different groups (**A**); control (CON), (DMSO), olive oil (OVO), ellagic acid (EA), hepatocellular carcinoma model (HCC-M), and the HCC model treated with EA (EA + HCC). Scale bar = 50 µm (**B**) bar graph representing the area percent of immunostained cells in all groups. Data are presented as mean + SD. The mean difference was significant at level = 0.05; (**) significantly different from the HCC-M group at *p* ≤ 0.05; (*) significantly different from the CON group, *p* ≤ 0.05.

HCSCs were traced using CD44. The area percent of immunostained cells with CD 44 in group HCC-M sections was significantly higher than all other groups (*p* ≤ 0.05). On the other hand, such cells were markedly decreased in group EA + HCC compared to the untreated group HCC-M (*p* ≤ 0.05) (Figure 6A,B).

#### 3.1.4. qRT-PCR

**Figure 7:**
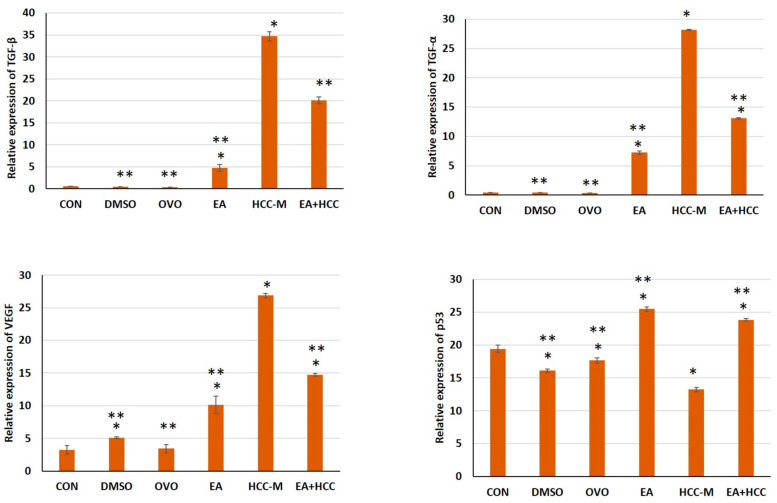
Bar graphs representing the relative expression of TGF-α, TGF-β, VEGF, p53 at m RNA level following the analysis of quantitative real-time PCR (qRT-PCR) in livers of all groups. Control (CON), (DMSO), olive oil (OVO), ellagic acid (EA), hepatocellular carcinoma model (HCC-M), and the HCC model treated with EA (EA + HCC). Data are presented as mean + SD. The mean difference was significant at level = 0.05; (**) significantly different from the HCC-M group at *p* ≤ 0.05; (*) significantly different from the CON group, *p* ≤ 0.05.

The effect of EA on TGF-α, TGF-β, VEGF, and p53 was studied in liver tissue of HCC-M and under therapy. EA had a mitigating effect revealed by downregulation of the elevated expression of TGF-α, TGF-β, and VEGF in HCC-M. On the other hand, EA restored the diminished expression of p53 in HCC-M, revealed by its upregulation in the EA + HCC group (Figure 7).

## 4. Discussion

It has been documented that inflammation plays a pivotal role in initiating tumor formation and how it advances throughout the stages [28]. In that regard, the present study aimed to assess the effect of EA on the inflammatory microenvironment of HCC in vivo. 

Based on previous studies, CCL4 was chosen to induce HCC in a rat model. CCL4 was related to the disturbance of amino acid and lipid metabolism [29,30]. Moreover, it was revealed that CCL4 can activate Kupffer cells, causing their hypertrophy to ultimately secret proinflammatory cytokines [31,32].

It has been reported previously that changes in organ weight are a sensitive indicator of drug toxicity, and it even proceeds any morphological alteration [33]. The HCC Aggressiveness Index (including the index for GGTP, bilirubin, and albumin) was positively correlated with the liver index [34]. Similarly, in the present study, the liver index scores for group HCC-M were significantly higher compared to all other groups (*p* ≤ 0.05). This might be due to lipid deposition, cell damage resulting in congestion, or cellular hyperplasia, as explained previously [35]. EA administration reduced such scores by 10.93%. 

Liver injury was indicated by seepage of transaminases, ALT, and AST and their elevation in the sera, which could be attributed to cell damage and plasma membrane breakdown. Other authors [36] confirmed such an explanation and proposed that such leakage occurs due to necrosis secondary to apoptosis, either as direct injury to hepatocytes or inflammation. On the other hand, it was also suggested that such an increase could be an induction of expression [37].

The increase in levels of ALP might be due to compression of the bile canaliculi with distorted ballooned hepatocytes. This aligns with what was explained that increased levels of ALP mark intrahepatic or extrahepatic obstruction of bile flow [38].

Tumor markers, AFP, and GGT confirming the HCC development in rats were evaluated. It was demonstrated that a high level of AFP indicates the severity of HCC [39]. AFP is even utilized as a prognostic marker following hepatectomy [36]. However, it was suggested that AFP is not discriminative enough to be a prognostic marker [40]. Hence, the serum gamma-glutamyl transferase (GGT) level was introduced as a biomarker associated with HCC. Furthermore, high GGT levels corresponded to poor clinical and pathological signs in HCC patients [41]. 

Histopathological examination of livers in group HCC-M revealed marked distortion of the hepatic structure correlated with the change in weight and biochemical evaluation of liver injury tests. The pleomorphism of the hepatocytes and their nuclei, the increase in nuclear-cytoplasmic ratio, and the appearance of oval cells strongly confirmed the development of HCC. The described structure agreed with previous results [42,43]. Interestingly, hepatic oval cells were correlated with the level of tissue damage and inflammation. They can also signify the clinical prognosis of liver diseases [44], as AFP is also considered a marker for oval cells [45]. 

Both tumor and cancer stem cells can acquire drug resistance, which may be attributed to resistance to apoptosis (extrinsic or intrinsic pathways), excessive repair of DNA damage by drugs, or multi-drug resistance [46,47]. 

In the present study, HCSC recorded a significant increase in the area immunostained by CD44 in the HCC-M. However, in response to EA therapy, a marked decrease was recorded. The importance of CSCs was reported earlier [48], who explained that CSCs play a pivotal role in the progress and severity of carcinogenesis, characterized by self-renewal, proliferation, sending metastasis, and resistance to drug therapy. Previously, it was mentioned that an increase in hepatic stem cell protein expression of cytokeratin 19, CD133, and CD44 is closely associated with tumor angiogenesis and poor prognosis [49]. Notably, in HCC, cytokine IL-6 released by tumor-associated macrophages can enhance the expansion of CD44+ CSCs [50]. Moreover, such cells can secrete TGF-β which can induce epithelial-mesenchymal transition in HCC by promoting properties of CSC [51].

The role of TGF-α and TGF-β in malignancy was investigated in earlier studies. 

Overexpression of TGF-α was linked to angiogenesis and metastasis of esophageal squamous cell carcinoma [52] and invasive breast cancer [53]. In HCC, overexpression of TGF-α induced proliferation, hyperplasia, and dysplasia [54]. 

Interestingly, TGF-β can play a dual role in HCC; in the earlier phases it enhances the anti-tumorigenic activity, but during the progression of the carcinoma, it switches to become a pro-tumorigenic factor [55]. Moreover, it was found to inhibit the cells of the innate immune system [11].

Regarding the role of transforming growth factors (TGFs) in the inflammatory microenvironment, previous studies proposed that a quiescent immune state (in the absence of inflammatory exciters) TGF-β is released, which stimulates the production of regulatory T cells. However, TGF-β in the presence of IL-6 (produced in acute stages inhibiting the regulatory T cells) induces the T helper 17 (Th17) cells. Th17 cells are considered proinflammatory cells and are then differentiated, boosting inflammation and halting autoimmune status [56]. 

Likewise, TGF-α stimulation was found to activate NF-κB under stress status directly [57,58]. It is established that NF-κB stimulates proinflammatory genes, establishes the function of effector T cells [59,60], and controls the efficacy of inflammasomes [61]. 

Much research focuses on the role of immunity in the therapeutic management of cancer [62].

In a deep second-degree burn rat model, it was recently discovered that EA successfully enhanced the healing process by adjusting the levels of inflammatory factors, increasing the immunoglobulin, IgA, IgG, and IgM, and the CD4+/CD8 + T cell ratio [63]. 

This confirmed previous results where the researchers tested the effect of EA on the HBe Ag secretion in a hepatitis B virus infection in vivo and in vitro. Their results revealed that the immune tolerance caused by HBe Ag was ameliorated by EA [64].

Based on the previous studies, TGF-α and TGF-β play a role in inflammation and tumorigenesis. The increased expression of TGF-α, and TGF-β, at the mRNA level in HCC-M confirmed such results. This result also reflects the therapeutic role of EA as it decreased the expression of both transforming growth factors in the treated group EA + HCC. 

The present findings revealed increased expression of VEGF at the mRNA level in the livers of HCC-M, which was significantly reduced on treatment with EA. In that regard, concentrations of VEGF were proved to increase proportionally to tumor size and severity of the HCC stage [65]. 

Moreover, HCSCs preferentially secrete exosomes to foster VEGF secretion from endothelial cells, which ultimately enhances tumor angiogenesis [66].

The present results aligned with earlier findings revealed the downregulation of p53 expression in HCC [67]. The absence of an effective form of p53 is essential for the induction of growth and proliferation of tumor cells in most types of cancers, including HCC [68]. It is noteworthy that P53, as a tumor suppressor gene, exhibited its effect mainly through activating apoptosis [69]. It is proved that the caspase family is the core mechanism for cell apoptosis in which the cleaved caspase 3 is the execution enzyme of apoptosis [70]. Such facts were confirmed in the present study by the simultaneous restoration and upregulation of p53 expression and the increase in caspase 3 immunostained cells in the group treated with EA + HCC. 

An interesting finding in the present work was the elevated expression of TGF-α, TGF-β, VEGF, and P53 under the effect of EA alone. Several causes were proposed previously [71]; the interaction between EA and different compounds of food may induce physiochemical interactions interfering with EA effects. Furthermore, co-ingestion of food contaminated with members of cytochrome P450 superfamily CYP1A1- or CYP3A4- can disturb the drug bioavailability. The authors also used a dose of 72 mg kg^−1^ day^−1^ of EA, which is equivalent to 813 mg given to a person weighing 70 kg. This also indicates that the effects of EA can vary according to the dose used, taking into consideration that the dose used in the current study was 50 mg/kg b.wt following previous experimental research. 

Other researchers added that the effects of the polyphenols could depend on the cell type and whether the cells are derived from the same or different tissue [72].

## 5. Conclusions

In conclusion, the anti-inflammatory, proapoptotic, anticarcinogenic effects of EA on HCC were evident by declining hepatic injury markers, ALT, AST, and ALP, tumor biomarkers, AFP and GGT, and restoration of the distorted hepatic structure. At the mRNA level, transforming growth factors (TGF-α and TGF-β) expression was reduced under treatment of EA with subsequent downregulation of VEGF and upregulation of p53. Ultimately, the upregulated p53 induced the execution of caspase 3 and targeted the HCSCs. In conclusion, the anti-inflammatory, proapoptotic, anticarcinogenic effects of EA on HCC were evident by declining hepatic injury markers, ALT, AST, and ALP, tumor biomarkers, AFP and GGT, and restoration of the distorted hepatic structure. At the mRNA level, transforming growth factors (TGF-α and TGF-β) expression was reduced under treatment of EA with subsequent downregulation of VEGF and upregulation of p53. Ultimately, the upregulated p53 induced the execution of caspase 3 and targeted the HCSCs. Further research for the detailed effect of EA on the liver can be studied by following the EA metabolites in the specific tissue.

## Figures and Tables

**Table 1:** List of primer pairs used in qRT-PCR (forward and reverse). The sequence is listed in the 5′ to 3′ direction.

	Forward	Reverse
P53	5′GTT CCGAGA GCT GAA TGA GG 3′	5′ TTT TAT GGCGGG ACG TAG AC3′
TGF alpha (TGF-α)	5′AAACACACGAGACGCTGAAG3′	5′ATCCAGTGAGTTCCGAAAGC3′
TGF beta TGF-β	5′TACCTGAACCCGTGTTGCTCTC3′	5′GTTGCTGAGGTATCGCCAGGAA3′
VEGF	5′CAG CTA TTG CCG TCC AAT TGA3′	5′CCA GGG CTT CAT CAT TGC A 3′
GAPDH	5′GCT CAC TAA AGG GCA TCC TG 3′	5′CCA TAGAGG CCA TGA GAT CC3′

**Table 2:** Effect of EA on sera levels of ALT, AST, and ALP in all groups; control (CON), DMSO, olive oil (OVO), ellagic acid (EA), hepatocellular carcinoma model (HCC-M), and HCC treated with EA (EA + HCC).

	CON	DMSO	OVO	EA	HCC-M	EA + HCC
ALT (U/L)	16.66 ± 0.18	17.7 ± 0.15 **	18.62 ± 0.13 **	19.40 ± 0.23 **	175 ± 8.39 *	27.50 ± 3.10 **^,^*
AST (U/L)	21.80 ± 0.83	23.60 ± 0.54 **	25.20 ± 0.83 **	30.40 ± 2.30 **	192.50 ± 9.34 *	33.60 ± 4.52 **^,^*
ALP (U/L)	45 ± 1.0	49.20 ± 0.83 **	65.20 ± 0.83 **^,^*	47.40 ± 2.30 **	195.10 ± 9.24 *	57.70 ± 2.71 **^,^*

Values are expressed as mean ± SD. (**) (*p* ≤ 0.05) statistically significant compared to the HCC-M group; (*) (*p* ≤ 0.05) statistically significant compared to the group CON.

## Data Availability

The data presented in this study are available in this article.
